# Requirements for a lead compound to become a clinical candidate

**DOI:** 10.1186/1471-2202-9-S3-S7

**Published:** 2008-12-10

**Authors:** Franz F Hefti

**Affiliations:** 1 Avid Radiopharmaceuticals, Inc., Market Street, Philadelphia, Pennsylvania 19104, USA

## Abstract

A drug candidate suitable for clinical testing is expected to bind selectively to the receptor site on the target, to elicit the desired functional response of the target molecule, and to have adequate bioavailability and biodistribution to elicit the desired responses in animals and humans; it must also pass formal toxicity evaluation in animals. The path from lead to clinical drug candidate represents the most idiosyncratic segment of drug discovery and development. Each program is unique and setbacks are common, making it difficult to predict accurately the duration or costs of this segment. Because of incidents of unpredicted human toxicity seen in recent years, the regulatory agencies and public demands for safety of new drug candidates have become very strict, and safety issues are dominant when identifying a clinical drug candidate.

## Introduction

Modern drug discovery and development efforts typically emerge from basic research and then gradually move on to specific sequential tasks, which – if successful – culminate in a new drug for the treatment of a human disease. The overall pathway is structured by well-delineated milestones, which include selection of the drug target, identification of a lead compound, its modification to a compound suitable for toxicity testing in animals, and selection as drug candidate for clinical testing. Although the road is well mapped out, it is by no means easy or guaranteed to end in success. Even before the onset of human studies, a drug candidate suitable for clinical testing is expected to satisfy specific and demanding criteria. It must bind selectively to the receptor site on the target and elicit the desired functional response from the target molecule. It must have sufficient bioavailability and distribution within the body to reach the receptor site, and it must elicit the desired responses *in vivo*, in animal models of the human disease. Most importantly, a drug candidate suitable for testing in humans must pass a formal toxicity evaluation in animals, to demonstrate that humans participating in the clinical studies are exposed to minimal risks only.

For many decades, knowledge and experience about drug discovery and preclinical drug development have been safeguarded by the pharmaceutical companies, and there was little outside interest. More recently, with the growth of the biotechnology industry and its open interface to academic research, drug discovery and development have become widely recognized as important, unique, and challenging activities.

## From lead molecule to a drug candidate suitable for testing in formal animal toxicity studies

The broad availability of chemical compound libraries and automatic screening technologies has made it relatively easy to identify initial lead candidates for new drug targets [1]. High quality leads are compounds that already satisfy some of the criteria for a future drug candidate. Chemists at Pfizer developed the so-called rule of five for leads and drug candidates [2]. The rule stipulates that molecules must have a molecular weight less than 500 g/mol, a partition coefficient (logP – a measure of hydrophobicity) less than 5, no more than five hydrogen bond donors, and no more than 10 hydrogen bond acceptors. Modern sample collections contain many molecules that violate the rule of five. Keeping the molecular weight under 500 has become particularly difficult because broad synthetic efforts naturally tend to increase the size of the chemicals. However, most successful drugs have molecular weights below 500 and logP values below 5, and failed compounds tend to fall outside this range [3]. In addition to satisfying the rule of five, high quality leads will have appropriate selectivity for the intended target, rather than giving positive signals in different screening assays. High quality leads will be attractive from the point of view of synthetic chemistry and offer diverse routes for multiple chemical modifications. Optimally, there will already be useful information on pharmacokinetic properties, showing that they are within a reasonable range of bioavailability and half-life. From the neuroscience perspective, it is particularly useful to have information on blood-brain barrier penetration.

Selection of a lead compound is followed by the initiation of a synthetic medicinal chemistry program. Table 1 lists the properties expected from a molecule taken into the expensive and time-consuming formal animal toxicity studies, which precede testing in humans. The criteria are vague by necessity, because they are determined by the intended route of administration and human disease targeted. In broad strokes, a molecule must satisfy basic needs of future manufacturing and storage. It has to produce the desired pharmacologic effects on the target and in animal models of the disease, with a route of administration and frequency of dosing commensurate with practical use in humans. It cannot produce any obvious signs of toxicity that preclude use in humans. Each medicinal chemistry program is unique, because it must focus on the deficiencies of the lead molecules.

**Table 1:** Desired properties for drug candidate taken for evaluation in formal animal toxicity studies.

**Properties**	**Details**
Chemical properties	Stable molecule
	
	Nonproblematic synthesis with potential for scale-up

Pharmacological properties	Selective high-affinity binding to target binding site
	
	Selective and potent functional effect on target receptor molecule *in vitro*
	
	Effectiveness in animal model of targeted human indication

Pharmacokinetics	Adequate bioavailability for selected route of administration
	
	Adequate half-life and biodistribution for intended use

Safety and toxicity	Satisfactory profile for inhibition and induction of cytochrome P450 enzymes
	
	Absence of obvious cardiac toxicity (hERG binding)
	
	Absence of obvious toxicity in animal studies

Each individual deficiency in efficacy, specificity, pharmacokinetic properties, and toxic potential must be addressed by defining the structure-activity relationship for the binding site that mediates an effect, and by synthesizing analogs that avoid the undesired binding sites. Each step taken and each deficiency resolved will affect other properties, making it impossible to address them in simple sequential order. Nevertheless, in a medicinal chemistry program, the selectivities of the leads are typically addressed first. Leads are evaluated in a broad counter-screening program, in which they are tested for affinity to most of the known drug receptor sites. These tests typically identify a small number of undesired binding affinities that must be worked out of the compound through chemical modifications. Counter-screen assays for the undesired affinities in a lead compound often remain an issue for the subsequent series of chemical derivatives, and they become part of the routine assays used in support of furthering the medicinal chemistry program.

Achieving adequate affinity to the receptor site is the next main goal of the medicinal chemistry program. To reach sufficient selectivity and potency *in vivo*, it is necessary to find compounds with binding affinities in the low nanomolar or even picomolar range. Binding assays are relatively simple and highly suitable for determining the affinity. Compounds with sufficiently high affinity move on to further assays that determine the functional effects on the target molecule, in order to distinguish agonistic from antagonistic responses. Binding and functional assays define the *in vitro *segment of the medicinal chemistry selection process. Compounds with sufficient binding affinity, selectivity, and functional efficacy are taken forward into further *in vivo *selection processes.

The *in vivo *assays serve to select compounds with satisfactory pharmacokinetic properties to reach the receptor site and to elicit the desired effect in animal models of the disease. There is a high degree of variability among drug discovery projects in the specific way in which this is handled. In the simplest possible case, a single animal model can satisfy all of these needs. The biologic efficacy of a compound in a predictive animal model after oral administration implies sufficient bioavailability and receptor occupancy, as well as appropriate effects on the receptor. For most drug discovery projects, however, these different steps are evaluated with individual assays. Standard pharmacokinetic methods are used to measure bioavailability and to select compounds with satisfactory properties. *In vivo *receptor occupancy assays determine adequate binding to the receptor. Finally, a functional model of the disease serves to measure the appropriate biologic response. The level of difficulty and capacity of the available *in vivo *models drive these decisions. Very often, animal models of specific diseases are complex and require treatment durations of several months. General comments on this topic would inappropriately blur and trivialize the large differences among drug discovery programs.

It is advantageous to incorporate early in medicinal chemistry programs assays that address specific safety concerns. Cardiovascular effects for example, even when minor, represent major problems for a drug candidate, because they could lead to sudden death in patients. Of particular concern are drugs that increase the QT interval in the sequence of cardiac contractions, because they predict an increased risk for heart attack [4]. The *I*_Kr _ion channel is the key determinant of the QT interval, and unfortunately this channel has a binding site with significant structural overlap with many G-protein-coupled receptor drug-binding sites. Thus, *I*_Kr _receptor site binding assays – in particular the human gene product that forms the channel, namely hERG (human ether-à-go-go-related gene) – are often incorporated into routine *in vitro *counter-screening batteries. In a similar way, inhibition or induction of cytochrome P450 enzymes, which are responsible for the majority of metabolic transformations of drugs, are often monitored early with *in vitro *systems.

In every program it is possible to detect significant toxicity issues by careful observations of animals used in efficacy studies. Major behavioral changes in motor activity, excretory functions, and body colors indicate potentially problematic effects. Inspection of organs after necropsy often divulges hints about toxic effects in the absence of extensive histopathology studies. Such casual observations, as well as exploratory toxicity studies, can play a major role in a drug discovery project, because they help to avoid setbacks after the time consuming formal animal toxicity studies.

Drug discovery projects evolve with time in distinct patterns. During the early stages the biologic assays are adjusted and optimized. Redundant steps are omitted. Mature drug discovery programs have a well-established critical path of *in vitro *and *in vivo *biologic assays by which chemicals are selected. Minimizing and optimizing the steps of the critical path is one of the keys to success in drug discovery. An optimized critical path allows the drug discovery researcher to select the best compound at each step without redundancies. The critical path will change if new issues are encountered that need to be resolved. The selection process of an optimized medicinal chemistry program and critical path has similarity to the Darwinian selection process of biologic evolution. However, chemicals are not randomly generated as are the mutations in biology, but rather they are designed based on rational considerations. The path from target selection to identifying a drug candidate is long and complicated. In rare cases, a few hundred molecules are necessary to achieve success, but it may be necessary to synthesize several thousands of molecules before an acceptable drug candidate emerges. Several years and large groups of chemists and biologists are often necessary to achieve this goal.

The basic criteria listed in Table 1 are applicable for small organic molecules as well as for biologic drug candidates. The success of natalizumab in multiple sclerosis [5] demonstrates that antibodies can be useful for the treatment of neurodegenerative diseases. High specificity and long half-life are key advantages of antibodies. In recent years, small inhibitory RNAs have emerged as new and highly attractive biologic drug candidates. The sequential steps by which molecular biologic methods are used to improve the antibodies and small inhibitory RNAs are analogous to those of a medicinal chemistry program. The critical path of *in vitro *and *in vivo *assays is closely similar; selectivity, potency, and efficacy must be established in the selection of the final drug candidate.

## Requirements for drug candidate suitable for human studies

In all countries, clinical studies with experimental drugs require the approval of regulatory agencies, who exercise substantial and dominant control over drug testing requirements and ultimate approval. In the USA regulatory oversight is provided by the Food and Drug Administration (FDA), whose activities are governed by law. The FDA requires filing of a Notice of Claimed Investigational Exemption for a New Drug (IND) for approval to proceed to clinical studies with a new drug candidate. The IND application describes the composition and synthesis of the drug candidate, and the data from *in vitro *studies and animal experimentation, as well as the clinical plans. The criteria used in the evaluation are explained in various publicly available guidance documents [6]. The essential features are listed in Table 2. Approval of an IND makes it possible to proceed to clinical testing of a drug candidate and is justifiably considered a very significant milestone in drug development.

**Table 2:** Information needed to support request for clinical testing of a drug candidate (IND filing to FDA).

**Information**	**Details**
Chemistry, Manufacturing and Control (CMC)	Compound with acceptable stability and formulation
	
	Controlled production under cGMP (current Good Manufacturing Processes)

Absorption, distribution, metabolism and excretion (ADME)	Route of administration, half-life
	
	Metabolic pathways
	
	Potential drug-drug interactions (including effects on cytochrome P450 enzymes)

Toxicology	Systemic and organ toxicity: gross and microscopic changes; two animal species, covering time periods of intended human exposure
	
	Estimated safety window between efficacious dose and 'no observed adverse effect level' (NOAEL)
	
	Initial data on potential genotoxicity and cardiotoxicity

Mechanism of action and pharmacology	Effects on receptor *in vitro*
	
	Efficacy in animal models *in vivo*

Clinical development plans	Detailed protocol of initial studies

FDA, Food and Drug Administration; IND, Notice of Claimed Investigational Exemption for a New Drug.

Minimizing the risk to participants in clinical studies requires that drug candidate material be manufactured, stored, and delivered according to highest specifications. Accordingly, these activities, which are generally referred to as Chemistry, Manufacturing, and Control (CMC), are intensely regulated. Adherence to standardized and regularly updated Good Manufacturing Practices is mandatory. A detailed description of these requirements would be beyond the scope of this short review and can be found in various regulatory guidance documents [6]. In essence, they ensure that the drug candidate is being produced in reliable and reproducible ways, where eventual impurities are characterized and shown not to be toxic, and that the material can be stored and delivered without degradation. Academic units and small biotechnology companies typically outsource CMC activities to specialized companies, whereas the large pharmaceutical companies have intramural units that specialize in these tasks.

To plan human studies and to minimize risk, it is necessary to make plausible predictions about the fate of the drug candidate in the human body after administration. Relevant predictive information, typically referred to as Administration, Distribution, Metabolism, and Excretion data, must obtained in animal studies before administration to humans. Initial information on the pharmacokinetic behavior of lead compounds and derivatives is typically obtained earlier in the medicinal chemistry program. For submission of the IND, these exploratory studies must be supplemented by results from more formal studies. Pharmacokinetic parameters – including bioavailability after oral or other systemic administration, plasma half-life, volume of distribution, clearance, and exposure – are determined in two or three animal species. Using established interspecies conversation factors, these data are than used to predict the human parameters.

Before use of a drug candidate in humans, it is necessary to characterize its metabolic transformations in the body. The vast majority of small organic molecules undergo metabolic transformation through hydroxylation and dealkylation, as well as conjugation reactions that covalently link the drug to naturally occurring hydrophilic compounds. Metabolic transformation of drugs sometimes generates unexpected toxic products. In every drug discovery project it is thus necessary to characterize the major metabolites and to assess their potential toxicity. Metabolic transformation determines the potential of a drug candidate to interfere with the function of another drug. Because drug-drug interactions often determine the success of a drug in medical practice, it is necessary to understand in detail their effects on metabolic enzymes in addition to the study of their own transformation. Metabolic transformations vary from molecule to molecule and can vary from species to species, often rendering their study a very complex and difficult task.

Formal toxicity studies in animals are the most important component of the characterization of a new drug candidate. They have to be carried out following the procedures of Good Laboratory Practice. The time period of administration to animals must exceed the intended treatment duration in humans. Plasma levels must reach sufficiently high levels to yield adequate high exposure to the tested drug candidate. Typically, during the course of the safety studies, toxic effects become apparent at the higher dose levels. These easily observable high-dose effects then guide the investigators in the search for less obviously toxic effects at lower doses. Besides behavioral observations, body fluid composition is analyzed for abnormalities and, at the end of the study, the organs of the test animals are weighed and the tissues processed for histological analysis. The toxicity studies provide an initial definition of the therapeutic window for a drug candidate. A 20-fold or higher difference is typically desired between clinically effective plasma levels in humans and those producing toxicity in animals. In addition, before clinical studies, all drug candidates must be evaluated in the standard *in vitro *tests predicting genotoxicity. Toxicity testing is the most unpredictable and most frustrating step in drug discovery and development. Most pharmaceutical companies share the experience that about three out of five small molecule drug candidates fail in animal toxicity testing for unpredictable and unknown reasons. Failure of a drug candidate in toxicity testing may set a program back by several months if not years, because new analogs without toxicity must be identified. To minimize this risk, experienced pharmaceutical companies typically generate several backup compounds in each medicinal chemistry program, with the hope that one among them will be devoid of toxicity.

The high attrition rate in safety testing observed with small organic compounds emphasizes the main advantage of biologic drugs. Biologics are less likely to exhibit unpredictable off-target activities than small organic molecules of xenobiotic origin. In addition, peptides, proteins, and RNAs tend to have simpler and more predictable metabolic pathways and pharmacokinetic properties. Consequently, the failure rate of biologics in animal toxicity studies tends to be lower than that of small organic molecules. These advantages have been widely recognized and drive the multiple attempts to generate biologic rather than small organic molecule drug candidates.

Broad information on mechanism of action and pharmacology is typically available for a clinical drug candidate. Information on *in vivo *efficacy maintains the enthusiasm for the program through the years of ongoing clinical studies, before clinical data become available. However, efficacy data are less critical than safety and toxicity data for the actual IND submission. A simple set of animal studies, sometimes even a plausible rationale, are sufficient to support the pharmacology part of an IND submission. Similarly, the clinical plans provided must emphasize safety. An overall clinical investigational plan must be provided, but the focus should on the detailed design of the early human safety studies. Patient safety is the absolute and primary concern in drug discovery and development, and the medical oath of not doing harm applies just as rigorously for participating drug discovery researchers.

## Conclusion

**Figure 1:**
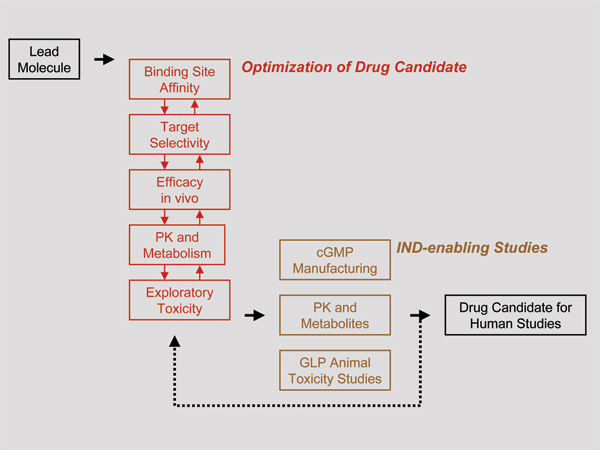
**Pathway from lead to drug candidate suitable for clinical trials**. The diagram represents the complex interactions, nonlinear nature of the process, and the potential setbacks. cGMP, current Good Manufacturing Practices; GLP, Good Laboratory Practice; IND, Notice of Claimed Investigational Exemption for a New Drug; PK, pharmacokinetics.

The path from lead to clinical drug candidate is not linear, even though it is often depicted as a sequence of modular activities and milestones. Figure 1 is an attempt to reflect the complex, modular interactions encountered in drug discovery projects. Each program is unique and tends to be idiosyncratic, defying generalization. Setbacks and failures occur frequently. Accordingly, the segment between lead and clinical drug candidate is often referred to as the 'zone of chaos', 'problem solving period', or 'valley of death' within the drug discovery and development pathway. To the great frustration of planners in funding agencies, universities, and companies, it is extremely difficult to predict accurately the duration or costs of this segment. On the positive side, it provides one of the most challenging and interesting of scientific activities [7].

Drug discovery programs are initiated and driven by the belief that an efficacious new drug can be identified and made available to suffering human patients. The positive signals and data for efficacy drive the program forward. In contrast, in the phase of selecting and characterizing a clinical drug candidate, the focus must be on human safety. Because of several incidents of unpredicted toxicity observed in human studies in recent years, the regulatory agencies and public demands for safety are stricter than ever before. Safety issues and concerns require the highest attention when identifying a clinical drug candidate.

## Competing interests

The author is an employee of and shareholder in Avid Radiopharmaceuticals Inc. (PA, USA) and shareholder of several biotechnology and pharmaceutical companies.

## References

[B1] Bajorath J (2002). Integration of virtual and high-throughput screening. Nat Rev Drug Discovery.

[B2] Lipinski CA, Lombardo F, Domini BW, Feeney PJ (1997). Experimental and computational approaches to estimate solubility and permeability in drug discovery and development settings. Adv Drug Deliv Rev.

[B3] Wenlock MC, Austin RP, Barton P, Davies AM, Leeson PD (2003). A comparison of physiochemical property profiles of development and marketed oral drugs. J Med Chem.

[B4] Redfern WS, Carlsson L, Davis AS, Lynch WG, MacKenzie I, Palethorpe S, Siegl PK, Strang I, Sullivan AT, Wallis R, Camm AJ, Hammond TG (2003). Relationships between preclinical cardiac electrophysiology, clinical QT interval prolongation and torsade de pointes for a broad range of drugs: evidence for a provisional safety margin in drug development. Cardiovasc Res.

[B5] Miller DH, Khan OA, Sheremata WA, Blumhardt LD, Rice GP, Libonati MA, Willmer-Hulme AJ, Dalton CM, Miskiel KA, O'Connor PW, International Natalizumab Multiple Sclerosis Trial Group (2003). A controlled trial of natalizumab for relapsing multiple sclerosis. N Engl J Med.

[B6] US Food and Drug Administration Center for Drug Evaluation and Research. Guidance Documents. http://www.fda.gov/cder/guidance/.

[B7] Hefti FF (2005). Drug Discovery for Nervous System Diseases.

